# Health insurance coverage for medical nutrition therapy in older women with cancer: a comparative health policy perspective on long-term care, frailty, and survivorship equity

**DOI:** 10.3389/fpubh.2026.1956760

**Published:** 2026-09-18

**Authors:** Jingqi Yang

**Affiliations:** 1 The University of Edinburgh, Edinburgh, United Kingdom; 2 Jiangsu Provincial University Key (Construction) Laboratory for Smart Diagnosis and Treatment of Lung Cancer, Kunshan, Jiangsu, China

**Keywords:** comparative health policy, frailty, health insurance, long-term care, malnutrition, medical nutrition therapy, older women with cancer, survivorship equity

## Abstract

Older women with cancer face overlapping risks of malnutrition, sarcopenia, frailty, treatment toxicity, functional decline, and dependence on long-term care. Medical nutrition therapy (MNT) can translate nutrition screening into individualized dietary counseling, symptom-responsive support, oral nutritional supplements, and enteral or parenteral nutrition when clinically indicated. Yet access is strongly conditioned by health insurance design. Eligibility restrictions, cost sharing, limited dietitian networks, fragmented authorization, and discontinuity between hospital, home, and long-term care can delay treatment until nutritional impairment is advanced. This targeted narrative Mini Review of selected high-income countries examines MNT coverage as a comparative health-policy and survivorship-equity issue for older women with cancer. We synthesize evidence linking nutrition impairment and frailty to quality of life, treatment tolerance, and survival; compare selected policy approaches in the United States, Australia, and the United Kingdom within broader universal health coverage and long-term care frameworks; and propose a conceptual, hypothesis-generating insurance-to-function framework linking coverage to access, nutrition care, and potential preservation of muscle reserve and independence. We propose that MNT be treated as an essential, risk-stratified component of oncology and long-term care rather than an optional wellness service. Proposed policy priorities include coverage triggered by validated nutrition and geriatric assessments, reimbursement for dietitian-led care and clinically indicated nutrition products, continuity across care transitions, integration with food and financial assistance, and equity-sensitive outcome monitoring.

## 1 Introduction

Population aging is increasing the number of women living with breast, gynecologic, colorectal, lung, and other cancers into later life. Global cancer incidence and mortality remain substantial, while longer survival shifts attention toward functional independence, treatment tolerance, and the affordability of supportive care (1). At the same time, universal health coverage remains incomplete: billions of people lack full access to essential services or experience financial hardship from out-of-pocket spending (2). These two transitions converge in older women with cancer, whose nutrition needs often extend beyond an acute treatment episode into rehabilitation, home care, and long-term care.

Medical nutrition therapy (MNT) is more than general dietary advice. It includes assessment by a qualified nutrition professional, diagnosis of nutrition problems, an individualized intervention, monitoring, and revision according to symptoms, treatment phase, comorbidity, function, and patient goals. In oncology, this may involve energy- and protein-dense counseling, management of nausea or dysphagia, oral nutritional supplements, and enteral or parenteral nutrition when oral intake is inadequate (3, 4). For older adults, nutrition is also a core domain of geriatric assessment and should be linked to management plans when impairment is identified (5).

For this narrative Mini Review, relevant literature and policy documents were identified through targeted searches of PubMed/MEDLINE and the official websites of the World Health Organization (WHO), the Organization for Economic Co-operation and Development (OECD), the Centers for Medicare & Medicaid Services (CMS), the Australian Government, Services Australia, and the National Institute for Health and Care Excellence (NICE) through 3 August 2026. Search terms combined medical nutrition therapy, cancer, older adults, women, frailty, malnutrition, health insurance, food insecurity, financial toxicity, survivorship, and long-term care. We prioritized clinical guidelines, systematic reviews, trials, observational studies, and current policy documents directly relevant to the review question; preprints and unpublished data were not used.

## 2 Why medical nutrition therapy matters in older women with cancer

Cancer-related inflammation, reduced intake, treatment adverse effects, multimorbidity, polypharmacy, and age-related anabolic resistance can jointly drive malnutrition, cachexia, and loss of skeletal muscle. European Society for Clinical Nutrition and Metabolism (ESPEN) and European Society for Medical Oncology (ESMO) guidance recommends early nutrition screening, repeated assessment, and escalation from counseling to oral, enteral, or parenteral support according to clinical need (3, 4). The Global Leadership Initiative on Malnutrition (GLIM) framework provides consensus diagnostic criteria for malnutrition, while Patient-Generated Subjective Global Assessment (PG-SGA)-based tools capture symptoms and intake barriers that are common during cancer treatment (6–8).

The prognostic signal is clinically important. In large cohorts of older patients with cancer, GLIM-defined malnutrition has been associated with worse survival and impaired quality of life, while nutritional impairment predicts poorer quality-of-life and psychological trajectories in advanced cancer (7–10). A recent systematic review found that trials in older patients remain heterogeneous, but nutrition interventions can attenuate decline in intake, weight, and muscle-related outcomes; an umbrella review similarly concluded that dietitian-provided interventions may improve nutrition status, intake, length of stay, and quality of life, although certainty is limited (11, 12).

Frailty modifies the consequences of nutrition impairment. Geriatric assessment-guided interventions reduce serious treatment toxicity, and randomized trials such as GAP70+, GAIN, and GERICO demonstrate the value of coordinated management of vulnerabilities that include nutrition, function, medication burden, and social support (13–15). Among surgical patients, frailty is associated with adverse postoperative outcomes, and geriatric comanagement can reduce mortality across frailty strata (16). For older women, osteoporosis, endocrine treatment, widowhood or living alone, lower lifetime earnings, and caregiving histories may further reduce nutritional and functional reserve. MNT coverage must therefore be designed around vulnerability rather than diagnosis alone.

## 3 Insurance coverage as a structural determinant of nutrition care

Insurance influences MNT through at least five mechanisms: eligibility, benefit scope, cost sharing, provider supply, and continuity. Narrow eligibility rules may exclude cancer unless a covered comorbidity is present. A benefit may reimburse counseling but not oral nutritional supplements or home enteral nutrition. Coinsurance can deter repeated visits, while low reimbursement and administrative burden can limit dietitian participation. Finally, authorization tied to a particular setting can interrupt care when a patient moves from hospital to home, rehabilitation, or a long-term care facility.

These design features matter because MNT is longitudinal. Nutrition risk changes with surgery, chemotherapy, radiotherapy, endocrine treatment, recurrent disease, and transition to palliative or long-term care. One-time counseling cannot substitute for monitoring of weight, intake, muscle function, treatment symptoms, and adherence. Cancer survivorship guidelines emphasize healthy dietary patterns, body-weight management, and individualized support, but implementation is uneven when professional nutrition care is not treated as a covered oncology service (17, 18).

Financial toxicity can also crowd out food expenditure. Food insecurity is recognized as an essential component of cancer care, and cost-related barriers may lead patients to forgo medical care or compromise diet quality (19, 20). Among older cancer survivors, persistent food insecurity is concentrated among women, people with lower income, racially marginalized groups, and those who are physically frail or experiencing anxiety and depression (21). Catastrophic health expenditures predict food insecurity in older survivors, while studies of women with cancer document the material and psychological burden of treatment costs (22–25). Insurance policy therefore affects nutrition both directly, through MNT coverage, and indirectly, through household resources available for food.

## 4 Comparative policy landscape: selected examples of coverage and fragmentation

In the United States, Medicare Part B covers MNT without cost sharing only for diabetes, kidney disease, or recent kidney transplantation; cancer alone is not an eligibility diagnosis (26). Enteral and parenteral nutrition can be covered under the prosthetic device benefit, but beneficiaries generally face deductible and coinsurance requirements (27). Newer Medicare services for community health integration and principal illness navigation can address food access and care navigation, including for cancer, but they do not substitute for a comprehensive cancer-specific MNT benefit (28, 29).

Australia’s chronic condition management framework supports structured multidisciplinary care and access to allied health services, including dietitians, under a general practitioner chronic condition management plan. Reforms implemented from July 2025 sought to simplify plans, strengthen continuity, and formalize referral processes, but the limited annual number of subsidized allied health contacts must be shared across disciplines and may be insufficient for complex oncology needs (30, 31).

In the United Kingdom, NICE guidance provides a clinical framework for oral, enteral, and parenteral nutrition in hospital and community settings, including multidisciplinary monitoring and individualized care plans (32). The practical availability of dietitians and community nutrition teams nevertheless varies locally. At the global level, WHO’s long-term care package and current access guidance call for integrated health and social services within universal health coverage and emphasize financial protection, while OECD data show wide cross-country variation in public long-term care spending and protection from unaffordable costs (33–36). These examples indicate that nominal coverage is not enough; effective coverage requires timely referral, workforce capacity, continuity, and protection from out-of-pocket costs.

## 5 The proposed insurance-to-function framework

The insurance-to-function pathway proposed here is a conceptual, hypothesis-generating framework (Figure 1); the complete pathway has not been empirically demonstrated. More generous and clearly defined coverage may lower financial and administrative barriers to nutrition and frailty screening, dietitian assessment, individualized protein-energy prescriptions, symptom management, and clinically indicated oral, enteral, or parenteral support. When earlier access is achieved, MNT may help stabilize intake, reduce cumulative energy and protein deficits, and support muscle, metabolic, immune, and wound-recovery processes. These potential effects concern supportive care and functional reserve rather than direct tumor biology.

**Figure 1:**
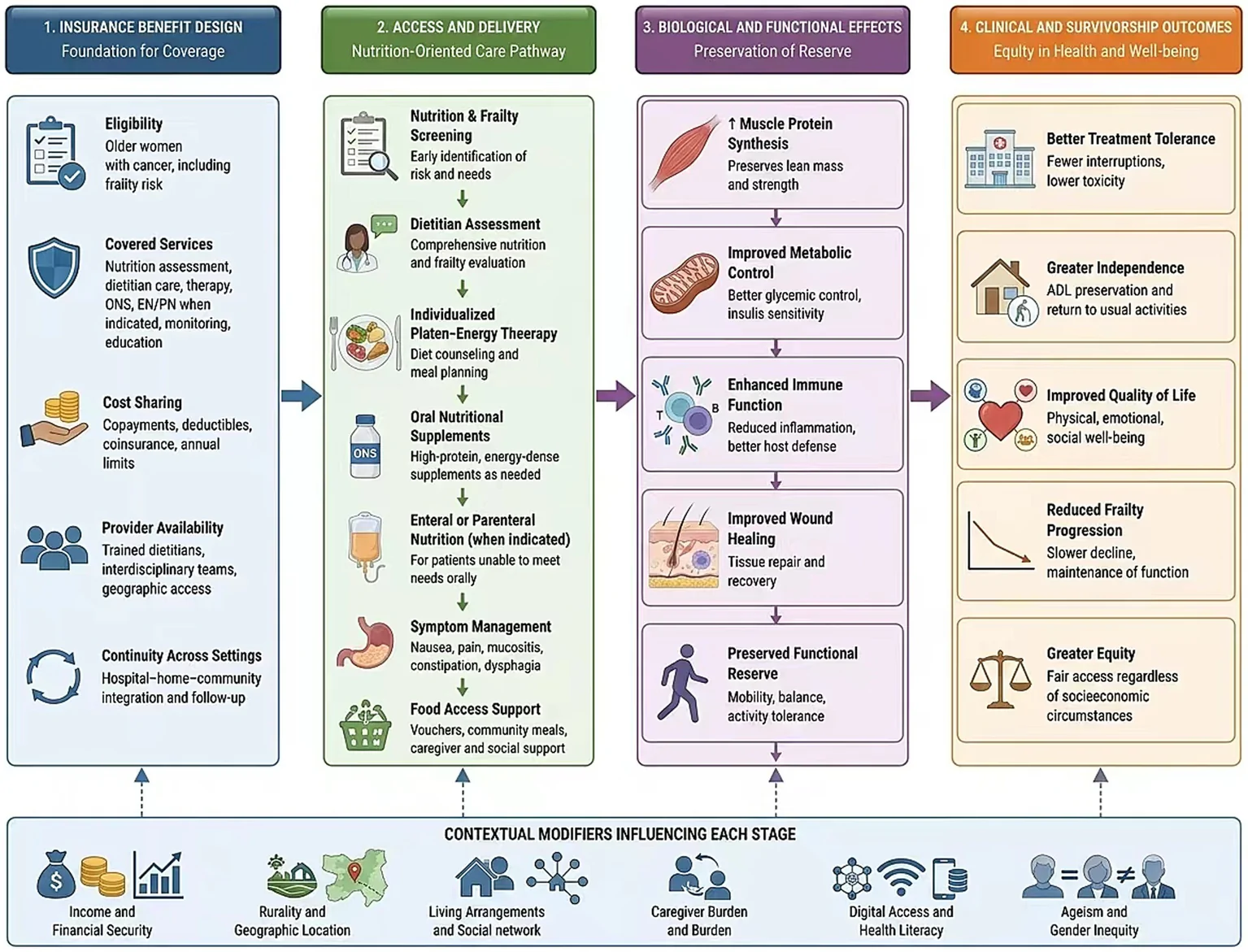
Conceptual insurance-to-function framework linking health insurance coverage for medical nutrition therapy with frailty and survivorship outcomes in older women with cancer. Benefit design determines eligibility, covered services, cost sharing, provider availability, and continuity across settings. These features influence access to nutrition and frailty screening, dietitian assessment, individualized protein-energy therapy, oral nutritional supplements, enteral or parenteral nutrition when indicated, symptom management, and food-access support. Timely treatment may preserve muscle protein synthesis, metabolic control, immune and wound recovery, and functional reserve, thereby supporting treatment tolerance, independence, quality of life, and equity. Income, rurality, living arrangements, caregiver availability, digital access, ageism, and gender inequity modify each stage. MNT, medical nutrition therapy; ONS, oral nutritional supplements; EN, enteral nutrition; PN, parenteral nutrition; ADL, activities of daily living.

The opposite pathway is equally important. When coverage is absent or difficult to use, screening may not trigger referral, patients may ration food or supplements, and follow-up can stop after discharge. Cross-sectional oncology surveys continue to show unmet nutrition needs and inconsistent engagement with registered dietitians, particularly after treatment and among people with symptom burden or limited support (37). Consensus recommendations for older adults in long-term care and community settings explicitly identify equitable access to MNT as a systems and insurance issue (38).

## 6 Why older women warrant an equity-specific lens

Older women are more likely than men to live alone, have lower pensions and savings, and rely on fragmented informal care after having provided unpaid care themselves. These conditions can reduce transport, digital access, food purchasing power, and the ability to manage complex home nutrition regimens. WHO’s social determinants framework emphasizes that gender, income, place, disability, and access to resources jointly produce avoidable health inequities (39).

The evidence is heterogeneous and should not be reduced to an assumption that all older women are food insecure. A national study found lower overall food insecurity among women with a history of breast cancer than women without cancer, but still recommended attention when financial problems are present (23). Conversely, rural women describe substantial financial toxicity, and racial inequities in cancer-related financial burden are well documented (25). The policy implication is targeted universalism: MNT should be routinely available, while intensity and ancillary benefits should be increased for those with frailty, low income, food insecurity, rural residence, limited caregiving, or high symptom burden.

Insurance coverage must also extend beyond clinic visits. Small trials suggest that cash transfers, medically tailored meals, pantry linkage, and food assistance can improve diet-related or food-security outcomes in oncology populations (40, 41). Such interventions are not replacements for MNT; they are implementation supports that allow a dietitian’s prescription to become feasible in daily life.

## 7 Policy priorities

### 7.1 Evidence-supported care priorities

Evidence-supported care priorities begin with validated nutrition screening and geriatric assessment to identify risk, followed, when indicated, by diagnostic evaluation for GLIM-defined malnutrition and separate validated assessment of sarcopenia and frailty, with timely dietitian assessment and repeated review (3–12).

Current clinical guidance supports a stepped care pathway from symptom-responsive counseling to oral nutritional supplements and, when indicated, enteral or parenteral nutrition, with monitoring across hospital, home, rehabilitation, and long-term care settings (3–5, 17, 18, 38).

### 7.2 Proposed policy innovations

Building on this evidence, we propose that MNT be treated as an essential, risk-stratified oncology and survivorship benefit rather than a diagnosis-limited preventive service. Potential innovations include eligibility based on clinical need, reimbursement for dietitian follow-up and clinically indicated nutrition products, caregiver education, tele-nutrition where appropriate, and continuity not constrained by arbitrary annual visit caps.

Additional proposed innovations include coordinated or bundled payment across care settings, equity-sensitive quality indicators, financial navigation, food-security screening, referral to transport, meal, or cash-support programs, and investment in the nutrition workforce (24, 40–42). These proposals require prospective implementation and economic evaluation.

## 8 Discussion and conclusion

The clinical case for nutrition assessment in older adults with cancer is stronger than the policy infrastructure that finances it. Malnutrition and frailty are common, measurable, and linked to treatment tolerance, quality of life, function, and survival, yet MNT coverage remains fragmented across diagnoses and care settings. Current evidence does not establish that broader insurance coverage alone improves cancer survival, and economic evaluations specific to older women are scarce.

Several limitations should be acknowledged. As a targeted narrative review, evidence selection is susceptible to narrative selection bias and was restricted to three selected high-income countries, limiting generalizability. Coverage policies may change over time, sex-specific evidence for older women remains limited, and no study identified directly tested the complete insurance-to-function pathway. Future research should compare benefit designs using natural experiments, claims data, patient-reported access, and pragmatic implementation trials. Studies should report sex-disaggregated outcomes, include long-term care and home-based populations, and distinguish nominal coverage from effective access. Economic evaluations should measure downstream hospital use, treatment completion, disability, caregiver burden, and quality-adjusted survival. Future comparative policy research should also include low- and middle-income countries, where informal care and out-of-pocket payment dominate.

In conclusion, health insurance coverage for MNT is a health-policy issue at the intersection of cancer nutrition, aging, and long-term care. For older women with cancer, equitable coverage should identify nutritional vulnerability early, finance evidence-based treatment, and maintain support across care transitions. The policy objective is not simply more nutrition visits; it is preservation of function, dignity, and meaningful survivorship without financial hardship.
